# Lowering blood cholesterol does not affect neuroinflammation in experimental autoimmune encephalomyelitis

**DOI:** 10.1186/s12974-022-02409-x

**Published:** 2022-02-07

**Authors:** Solenne Vigne, Donovan Duc, Benjamin Peter, Jessica Rebeaud, Yannick Yersin, Florian Ruiz, Valentine Bressoud, Tinh-Hai Collet, Caroline Pot

**Affiliations:** 1 Laboratories of Neuroimmunology, Neuroscience Research Center and Service of Neurology, Department of Clinical Neurosciences, Lausanne University Hospital and University of Lausanne, Chemin des Boveresses 155, 1066 Epalinges, Switzerland; 2 Service of Endocrinology, Diabetes, Nutrition and Therapeutic Education, Department of Medicine, Geneva University Hospitals (HUG), Rue Gabrielle-Perret-Gentil 4, 1211 Geneva 14, Switzerland

**Keywords:** Autoimmunity, Multiple sclerosis, EAE, Neuroinflammation, Cholesterol, Hypercholesterolemia, LDL receptor, PCSK9, Alirocumab

## Abstract

**Background:**

Multiple sclerosis (MS) is a chronic disabling disease of the central nervous system (CNS) commonly affecting young adults. There is increasing evidence that environmental factors are important in the development and course of MS. The metabolic syndrome (MetS) which comprises dyslipidemia has been associated with a worse outcome in MS disease. Furthermore, the lipid-lowering drug class of statins has been proposed to improve MS disease course. However, cholesterol is also rate-limiting for myelin biogenesis and promotes remyelination in MS animal models. Thus, the impact of circulating blood cholesterol levels during the disease remains debated and controversial.

**Methods:**

We assessed the role of circulating cholesterol on the murine model of MS, the experimental autoimmune encephalomyelitis (EAE) disease using two different approaches: (1) the mouse model of familial hypercholesterolemia induced by low-density lipoprotein receptor (LDLr) deficiency, and (2) the use of the monoclonal anti-PCSK9 neutralizing antibody alirocumab, which reduces LDLr degradation and consequently lowers blood levels of cholesterol.

**Results:**

Elevated blood cholesterol levels induced by LDLr deficiency did not worsen clinical symptoms of mice during EAE. In addition, we observed that the anti-PCSK9 antibody alirocumab did not influence EAE disease course, nor modulate the immune response in EAE.

**Conclusions:**

These findings suggest that blood cholesterol level has no direct role in neuro-inflammatory diseases and that the previously shown protective effects of statins in MS are not related to circulating cholesterol.

**Supplementary Information:**

The online version contains supplementary material available at 10.1186/s12974-022-02409-x.

## Background

Multiple sclerosis (MS) is a chronic inflammatory and autoimmune disease affecting the central nervous system (CNS) leading to neuronal damage and disabling neurological deficits [1]. It is a common disorder affecting young adults; its mortality is low, but it is a lifelong disease with high morbidity. The etiology of MS is multifactorial and environmental factors play a major role in disease causation [2]. In line with this concept, obesity, defined as a body mass index ≥ 30 kg/m^2^, is associated with increased risk of MS [3, 4]. Several studies have shown that obesity during childhood [5, 6] or adolescence [7, 8] promotes MS. Metabolic changes associated with obesity disrupt lipoproteins and their content and are often dubbed dyslipidemia, i.e., elevated total cholesterol (tChol), low-density lipoprotein cholesterol (LDLc), and triglycerides, and decreased high-density lipoprotein cholesterol (HDLc). This constellation has been associated with poor outcomes of MS [9–14]. It has been further proposed that cholesterol modulates the immune system and that hypercholesterolemia drives a proinflammatory response [15]. However, cholesterol is also indispensable in the CNS as it is a component of cellular membranes and myelin [16], is required for synapse and dendrite formation [17] and for axonal guidance [18]. Thus, the role of cholesterol metabolism during MS is largely debated and the underlying mechanisms remain unclear.

Initial studies have looked into the relationship between lipid metabolism and neuroinflammation by assessing the effect of statins, an enzymatic inhibitor of the 3-hydroxy-3-methylglutaryl coenzyme A reductase thus reducing cholesterol levels. Statins are the first-line drug class to lower cholesterol levels used in cardiovascular disease prevention and treatment. While statins dampen the severity of the MS mouse model, the experimental autoimmune encephalomyelitis (EAE), it has been later suggested that the beneficial effect of statins, could be independent of their cholesterol-lowering effects and related to their immunomodulatory activities similar to the ones observed with approved MS medications [4, 19–21]. Moreover, the beneficial effect of statins remains controversial as studies have led to contradictory results in MS and their different subsets, relapsing–remitting MS (RRMS) versus secondary progressive MS (SPMS) [22]. Hypocholesterolemia could on the other hand be deleterious for myelin formation and for myelin repair, a process that is beneficial in MS disease, at least in animal models such as in EAE and in the cuprizone model targeting more specifically demyelination [23–25]. Furthermore, sex hormones could modify immunomodulatory lipoprotein functions and the impact of lipoprotein may differ between female and male mice [26]. Indeed in mouse models, LDLr deficiency was shown to attenuate EAE disease severity only in female mice through the induction of apolipoprotein E (Apo E) [27] and it has been proposed that EAE disease is less severe in ApoE-deficient mice [28]. However, other studies have on the contrary shown that EAE disease was more severe in ApoE-deficient mice by promoting blood–brain barrier (BBB) permeability [29, 30]. Of note in the ApoE studies, the sex-specific effects of the animals could contribute to the different results observed [26, 31]. While a role of ApoE in EAE and MS had initially been suggested, the specific association between ApoE and a higher susceptibility risk for MS was not confirmed in a large-scale genome-wide association study (GWAS) [32]. Overall, it is not clear a) whether the elevated circulating cholesterol levels observed in MS patients promote inflammation; b) if elevated cholesterol levels could be needed for tissue repair in the CNS; c) and whether cholesterol-lowering drugs can be considered as a treatment in MS. Moreover, few studies have determined the exact contribution of altered lipid profiles and especially cholesterol in the progression of the disease. Thus, studies investigating the role of cholesterol in demyelinating diseases should be carried out to clarify the role of cholesterol in MS.

Recently, one of the greatest advances in clinical lipidology has been the development of monoclonal antibodies targeting the *Proprotein convertase subtilisin/kexin type 9* (PCSK9). Discovered in 2003, PCSK9 is a serine protease that promotes the intralysosomal degradation of the LDL receptor, resulting in reduced hepatic LDLc uptake and increased plasma LDLc concentrations. Monoclonal anti-PCSK9 neutralizing antibodies are currently used for a potent reduction of the LDLc levels by 50–60% and are indicated for patients with familial hypercholesterolemia or those who are statin-intolerant who need cardiovascular prevention. Their putative role has been explored in neurodegenerative disorders, in particular in Alzheimer’s disease (AD), where cholesterol pathways might also be involved [33]. The contribution of PCSK9 in AD pathogenesis is however controversial [34]. Moreover, the role of PCSK9 has not been studied during neuroinflammation, nor on EAE or during MS.

In the present work, we show that increasing or conversely reducing blood cholesterol does not alter neither the peripheral adaptive immune responses nor the immune cell infiltration of the CNS during EAE disease. This study provides new evidence that the sole lowering of circulating cholesterol might not be sufficient to target neuroinflammation. It further suggests that the beneficial effects of cholesterol-lowering drugs like statins in the treatment of EAE are associated with non-cholesterol-related processes rather than with the specific decrease of circulating cholesterol.

## Methods

### Animals

C57BL/6J and LDLr^−/−^ (C57BL/6J background, Jackson Laboratory, stock number: 002207) mice were bred in the animal facility at Lausanne University Hospital under specific-pathogen-free conditions. The animals had access to food and tap water ad libitum with a constant 12-h light/dark cycle. All mice were aged between 8 and 10 weeks. All procedures and methods were performed following guidelines from the Cantonal Veterinary Service of canton of Vaud, Switzerland.

### EAE induction and clinical evaluation

For induction of EAE, mice were immunized with 100 µg myelin oligodendrocyte glycoprotein (MOG) peptide 35–55 (MOG_35–55_) (Anawa) or PBS emulsified in complete Freund’s adjuvant supplemented with 5 mg/ml *Mycobacterium tuberculo*sis H37Ra (BD Difco). A total of 200 µl emulsion was subcutaneously injected into four sites on the flanks of mice. On days 0 and 2 after initial peptide injections, animals received an additional intravenous injection of 100 ng pertussis toxin (Sigma Aldrich). Mice were scored daily for clinical symptoms. The EAE symptoms were assessed according to the following score: score 0—no disease; score 0.5—reduced tail tonus; score 1—tail paralysis; score 1.5—impaired righting reflex; score 2—hind limb weakness; score 2.5—partial hind limb paralysis; score 3—Complete hind limbs paralyzed; score 4—forelimb paresis and complete hind paralysis; score 5—moribund or dead. Mice were euthanized if they reached a score > 3.

### Antibody treatment

For lowering cholesterol experiments, mice were intraperitoneally injected with 10 mg/kg of anti-PCSK9 (proprotein convertase subtilisin/kexin type 9) (alirocumab) or PBS control 1 week before EAE immunization and once per week until the end of the experiments.

### Quantification of lipid profile

Blood from mice was collected submandibular and serum was isolated using centrifugation. Serum lipid profiles were assessed using Roche Cobas C111 robot from the Mouse Metabolic Evaluation Facility (University of Lausanne, Switzerland) and Siemens Dimension Xpand plus from the Center of Phenogenomics (EPFL, Lausanne, Switzerland).

### Histology

Mice were killed at the end of the EAE disease and perfused with cold PBS followed by 4% paraformaldehyde fixation. Spinal cord tissue was embedded in paraffin. For light microscopy, sections were stained with hematoxylin and eosin (HE) or Luxol Fast Blue and periodic acid Schiff (LFB/PAS) (Sigma–Aldrich). Images of tissue sections were scanned using a Nanozoomer S60 slide-scanner. Inflammatory foci per spinal cord were quantified on HE-stained cross-sections. LFB/PAS staining was performed to assess demyelination, and the demyelinated area of spinal cross-sections (expressed as percentage of total white matter) was measured using the Fiji Image J software. Average values of five cross-sections per animal were calculated.

### Antigen-specific proliferative and cytokine responses

Single-cell suspensions were prepared from spleens 10 days post-immunization for EAE. Cells were restimulated with MOG_35–55_ for 72 h in supplemented DMEM medium containing inactivated 10% FCS (FBS 18, Biowest), 100 U/ml penicillin–streptomycin (BioConcept), 1 mM sodium pyruvate (Sigma), 50 M β-mercaptoethanol (Gibco), MEM non-essential amino acids (100×) (Gibco), MEM vitamins (100×) (Sigma), 200 mM l-glutamine, folic acid 14 mM (Sigma), 0.3 mM l-asparagine (Sigma), 0.7 mM l-arginine. For proliferation assays, cells were pulsed with 1 μCi of [3H]-thymidine (Hartmann Analytic) during the final 18 h and analysis of incorporated [3H]-thymidine was performed in a β-counter (Packard Top Count NXT Luminescence and Scintillation Counter). Secreted cytokines were measured after 48 h of culture with MOG_35–55_ by ELISA (Invitrogen).

### Isolation of immune cells

Mice were perfused through cardiac ventricle with phosphate-buffered saline (PBS) 1 ×. Brain and spinal cord were cut into pieces and digested for 45 min at 37 °C with collagenase D (2.5 mg/ml; Roche) and DNAse I (1 mg/ml; Roche) followed by 70%/37%. Percoll gradient (GE Healthcare) centrifugation. The cellular suspensions were washed and filtered through 40-µm cell strainers and resuspended in culture medium for further analysis.

### Flow cytometric analysis

Single-cell suspensions in PBS 1 × were stained with fixable viability dye eFluro™ 620 (eBioscience). Cells were preincubated with anti-CD16/32 for 10 min to block Fc receptors and stained in FACS buffer (PBS containing 1% BSA) with directly labeled monoclonal antibodies for 30 min. For intracellular cytokine staining, cells were activated for 4 h with 50 ng/ml PMA, 1 μg/ml ionomycin in the presence of 10 mg/ml brefeldin A. After surface staining, cells were fixed and permeabilized using Foxp3/transcription factor staining buffer set and stained intracellularly with directly labeled monoclonal antibodies for 30 min. Data were acquired on LSR II cytometer and all data were analyzed using FlowJo software. Fluorochrome-conjugated antibodies were purchased from several commercial sources indicated below. Antibodies against CD45 (30-F11) was from Biolegend; CD3 (145-2C11), CD4 (GK1.5), IL-17A (Q31-378), RORγt (ebio17B7), T-bet (ebio4B10) and IFN-γ (XMG1.2) were from eBiosciences.

### Statistical analysis

Data analyses and graphs were performed using GraphPad Prism 7.0 software. *p*-values < 0.05 was considered significant. *p*-values of cholesterol concentration, cytokine production, histology quantification and cell frequency were determined by unpaired Student’s *t* test or two-way ANOVA with Sidak’s post hoc test as detailed in the corresponding figure legends. The EAE clinical scores and the area under curve (AUC) were compared with the non-parametric Mann–Whitney *U* test. Results are displayed as mean and SEM, or mean and SD, as described in the figure legends.

## Results

### LDLr deficiency does not impact EAE disease progression

**Fig. 1:**
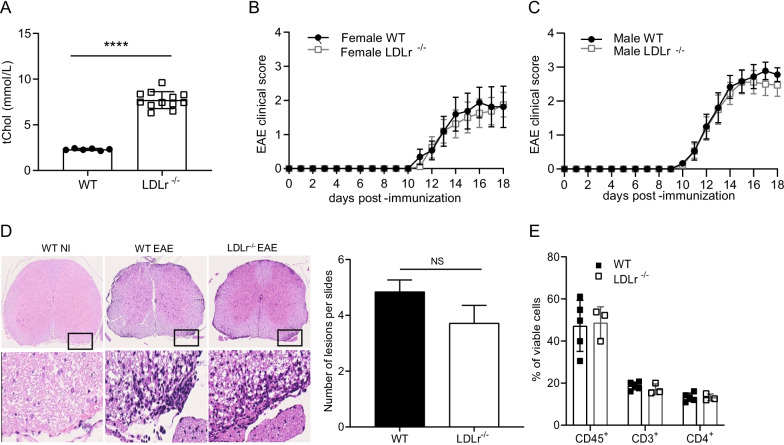
Hypercholesterolemia induced by LDLr deficiency do not exacerbate EAE disease. **A** Serum level of total cholesterol (tChol) in female LDLr^−/−^ (mean ± SD; *n* = 6) and wild-type mice (mean ± SD; *n* = 12 mice). **B** EAE in female wild-type and LDLr^−/−^ mice. The course of EAE is shown as clinical score (mean ± SEM; *n* = 8 mice). **C** EAE in male wild-type and LDLr^−/−^ mice. The course of EAE is shown as clinical score (mean ± SEM; *n* = 9 mice). The female and male EAE experiments were not performed at the same time. Data are representative of three experiments. **D** Histopathological stainings and quantifications of spinal cord sections of non-immunized WT (WT NI) or immunized WT and LDLr^−/−^ mice at day 16 post-immunization for cellular infiltration (H&E). Five sections per mouse were quantified (*n* = 3 mice). Scale bars 500 µm (top panels), 100 µm (bottom panels). **E** Flow cytometry analysis of the total proportion (%) of the leukocyte (viable CD45^+^), lymphocyte T (CD3^+^) and lymphocyte T CD4^+^ (CD4^+^) in the CNS 14 days after EAE immunization (mean ± SD; *n* = 3–5 mice). ∗∗∗∗*p* < 0.0001; NS, not significant; *p* values were determined by unpaired Student’s *t* test (**A** and **D**), a Mann–Whitney *U* test (**B**, **C**) and a two-way ANOVA with Sidak’s post hoc test (**E**).

A previous report described a protective role of LDLr deficiency in female but not in male EAE mice [27], however, the nature of the observed sexual dimorphism remains unclear. Here, we first evaluated female LDLr^−/−^ mice. We confirmed that LDLr^−/−^ mice exhibited higher blood cholesterol. Blood was collected from WT and LDLr^−/−^ female mice before induction of EAE. LDLr^−/−^ mice had a significant twofold elevation of total cholesterol compared to WT mice (Fig. 1A). To elucidate whether hypercholesterolemia impacts EAE severity, WT and LDLr^−/−^ female mice were immunized with MOG_35–55_ peptide. We observed a similar EAE disease course in both groups (Fig. 1B). The experiment was repeated in male mice to assess the sex-dependent effects of hypercholesterolemia on EAE severity. We observed that male LDLr^−/−^ mice exhibited similar EAE disease course compared to their respective WT group (Fig. 1C). We further evaluated the extent of CNS infiltrates in EAE diseased animals at the end of the experiment. No significant differences were observed between sick LDLr^−/−^ and WT mice concerning the number of inflammatory lesions (Fig. 1D) assessed by histology as well as by flow cytometry (Fig. 1E). In addition, we did not notice changes in demyelination assessed by LFB/PAS staining (Additional file 1: Fig. S1). In our settings, we did not observe any impact of LDLr deficiency on the development of EAE.

### LDLr deficiency does not influence the peripheral CD4^+^ T cell priming to MOG_35–55_

**Fig. 2:**
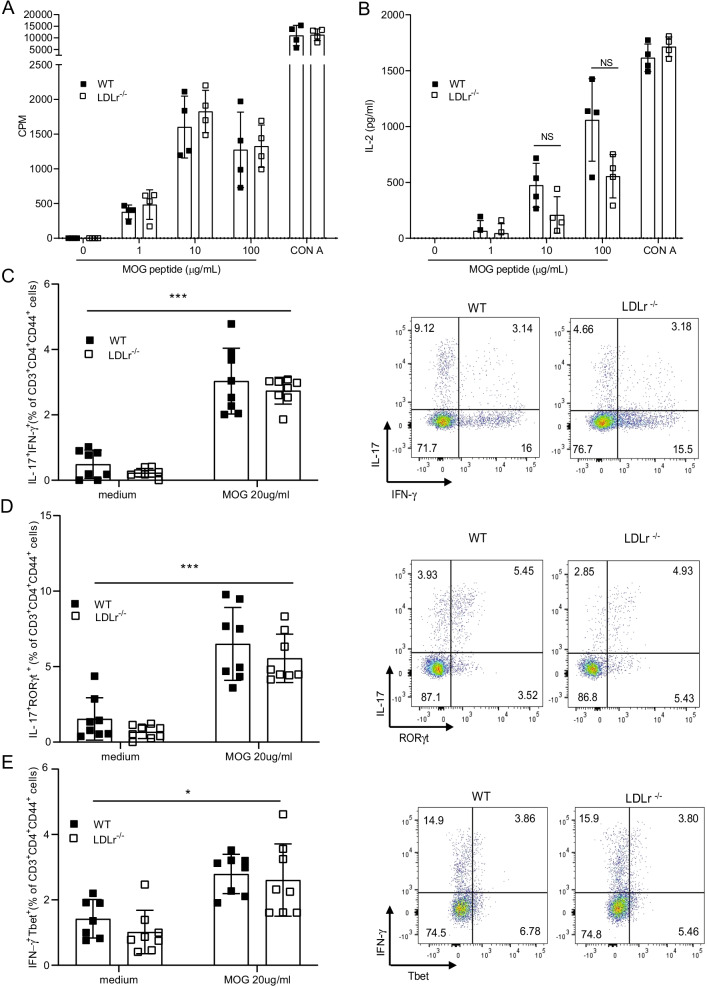
LDLr deficiency does not influence proliferation nor cytokine production induced by a T cell recall response. **A** On day 8 after immunization, splenocytes were isolated from WT and LDLr^−/−^ mice and restimulated with MOG_35-55_ in vitro. The proliferative response was measured by [3H] thymidine incorporation 72 h after restimulation with different concentrations of MOG_35-55_ peptide or Concanavalin A (CON A) and expressed in counts per minute (CPM) (mean ± SD, *n* = 4 mice). **B** Cytokine IL-2 production in culture supernatants after 48 h of culture with the indicated concentration of MOG_35–55_ or CON A at 10 µg/ml was determined by ELISA (mean ± SD, *n* = 4 mice). **C**–**E** Flow cytometric analysis of the percentage of IL-17^+^IFN-γ^+^,IL-17^+^RORγt^+^ and IFN-γ^+^Tbet^+^ in CD3^+^CD4^+^CD44^+^ T cell at day 6 after restimulation of the indicated concentration of MOG_35–55._ Representative dot plots for **C**–**E** are results from WT and LDLr^−/−^ splenocytes restimulated with 20 μg/ml of MOG_35–55_. Shown results are from mice pooled from two independent experiments (mean ± SD, *n* = 8 mice). Data are representative of three experiments. ∗*p* < 0.05, ∗∗∗*p* < 0.001, NS, not significant; *p* values were determined by two-way ANOVA with Sidak’s post hoc test (**A**–**E**)

Antigen-activated T cells are key effector cells in the pathogenesis of EAE, which are first activated in secondary lymphoid organs where they expand before migrating to the CNS. Moreover, T cells are dependent on cholesterol to proliferate [35]. We thus investigated the influence of LDLr deficiency on T cell responses in the immune periphery compartment during the preclinical stage of EAE. As we did not observe sex differences in EAE disease, we continued our analysis on female mice only as it is usually performed in EAE. WT and LDLr^−/−^mice were immunized with MOG_35–55_ emulsified in CFA and pertussis injections. After 8 days, splenocytes from both LDLr^−/−^ and WT mice were isolated and stimulated with MOG_35–55_ peptide in vitro. Proliferation and activation in response to MOG_35–55_ were assessed by thymidine incorporation and IL-2 secretion in the culture supernatants. No significant differences in T cell mitotic activities were observed between LDLr^−/−^ and WT splenocytes induced with MOG_35–55_ and CFA (Fig. 2A) and no significant changes in IL-2 levels in the supernatant were detected (Fig. 2B). Furthermore, we explored the frequency of IL-17A^+^IFN-γ^+^ CD4^+^ T cells, RORγt^+^IL-17A^+^ and Tbet^+^IFN-γ^+^ activated CD4 T lymphocytes (CD3^+^CD4^+^CD44^+^) after 6 days of culture with MOG_35-55_ by flow cytometry. Similarly, we observed that WT and LDLr^−/−^ mice display the same percentages of IL-17A^+^IFN-γ^+^ (Fig. 2C), RORγt^+^IL-17A^+^ (Fig. 2D) and Tbet^+^IFN-γ^+^ producing CD4 T lymphocytes (Fig. 2E). These data indicate that antigen-specific sensitization with MOG_35–55_ is not impaired in the absence of LDLr.

### Monoclonal anti-PCSK9 neutralizing antibody decreases circulating cholesterol without alleviating EAE symptoms

**Fig. 3:**
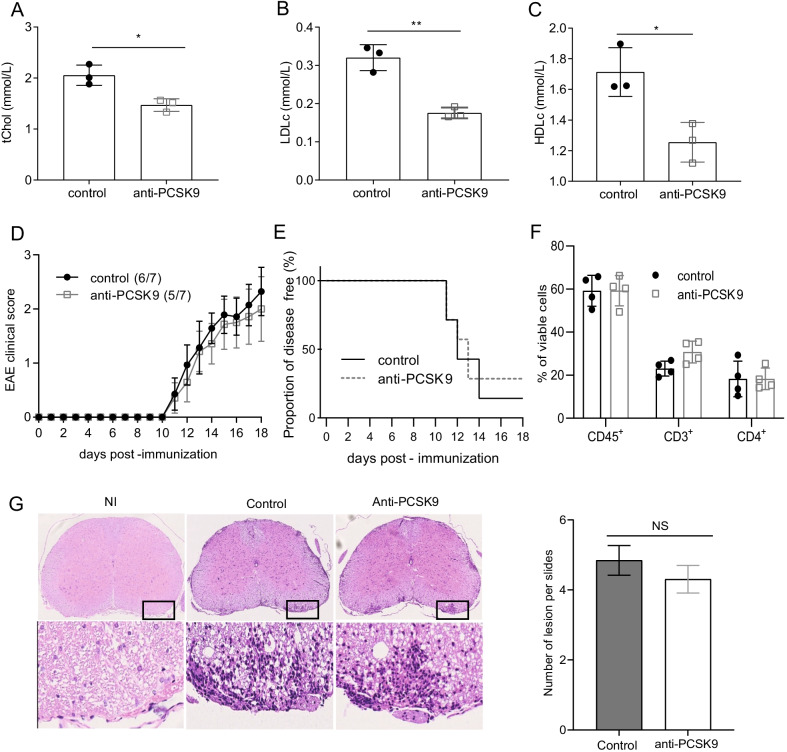
Anti-PCSK9 significantly decrease circulating cholesterol without alleviating EAE symptoms. **A** Quantification of circulating tchol, **B** LDLc and **C** HDLc of anti-PCSK9 treated mice versus PBS control group (mean ± SD, *n* = 3 mice). Data are representative of two experiments. Tchol, LDLc and HDLc levels were measured before anti-PCSK9 injection and 9 days after the first anti-PCSK9 injection **D** Clinical scores of EAE in immunized mice treated with anti-PCSK9 or PBS control (mean ± SEM, *n* = 7 mice). **E** Disease free activity between mice treated with anti-PCSK9 or PBS. **F** Flow cytometry analysis of the total proportion (%) of the leukocyte (viable CD45^+^), lymphocyte T (CD3^+^) and lymphocyte T CD4^+^ (CD4^+^) in the CNS 14 days after EAE immunization (mean ± SD; *n* = 4 mice). Data are representative of two experiments. **G** Histopathological staining and quantifications of spinal cord sections at day 16 post-immunization for cellular infiltration (H&E) of non-immunized (NI) or immunized WT mice treated with PBS as control or with anti-PCSK9. Five sections per mouse were quantified (*n* = 3). Scale bars 500 µm (top panels), 100 µm (bottom panels). ∗*p* < 0.05, ∗*p* < 0.01, NS, not significant; *p* values were determined by unpaired Student’s t test (**A**–**C** and **G**), and a Mann–Whitney *U* test (**D**) and a two-way ANOVA with Sidak’s post hoc test (**F**). AUC were compared by Mann–Whitney test (**E**)

As we observed that LDLr deficiency did not affect EAE disease progression, we further explored the inverse, whether reducing circulating cholesterol would attenuate the disease as described for statin treatment. Indeed, an effector mechanism responsible for this statin-mediated disease amelioration could be independent of their cholesterol-lowering properties. Therefore, we investigated the effects of a more selective class of lowering-cholesterol using the monoclonal antibody targeting circulating PCSK9 alirocumab, on the acute inflammatory responses and the progression of the disease. WT mice were treated either with anti-PCSK9 antibodies or PBS 1 week before EAE and once weekly until the end of the disease. We confirmed a significant reduction of circulating tChol, LDLc and HDLc in mice treated with anti-PCSK9 (Fig. 3A–C) compared to the PBS injected group. However, despite the decrease of serum cholesterol, mice developed EAE disease with similar severity (Fig. 3D) and similar incidence (Fig. 3E). EAE is featured by infiltration of activated lymphocytes into the CNS leading to a local inflammatory response. Immune infiltrates were assessed by flow cytometry and immunohistochemistry in the CNS at the peak of the disease. We did not observe significant differences in the number of immune cells infiltrating the CNS in mice treated with anti-PCSK9 versus control mice when assessed by flow cytometry (Fig. 3F). Similarly, the number of inflammatory foci assessed by histological analysis was similar in the two groups (Fig. 3G). Furthermore, anti-PCSK9 treatment did not significantly affect the percentage of demyelinated area during the acute phase of EAE in mice treated with anti-PCSK9 compared to PBS-treated WT and LDLr^−/−^ mice (Additional file 1: Fig. S1).

### Anti-PCSK9 treatment does not impair antigen-recall responses

**Fig. 4:**
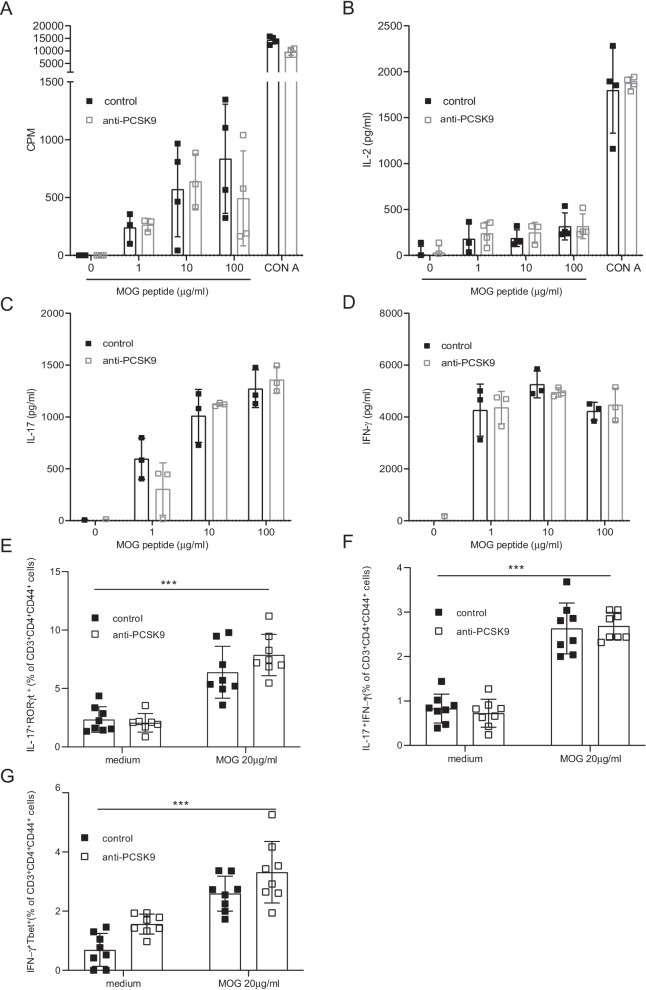
Anti-PCSK9 treatment does not show altered systemic immune responses. In vitro restimulation of splenocytes isolated from EAE immunized anti-PCSK9 treated mice versus control group with different concentrations of MOG_35-55_ peptide or CON A. **A** Proliferative response was determined by [3H]-thymidine integration and expressed in counts per minute (CPM) (mean ± SEM, *n* = 4 mice). **B**–**D** Cytokine production was determined by ELISA: Secretion of IL-2 (**B**), IL-17A (**C**) and IFN-γ (**D**) were measured by ELISA after 48 h of culture with the indicated concentration of MOG_35–55_ (mean ± SEM, *n* = 3–4 mice). **E**–**G** Flow cytometric analysis of the frequencies of IL-17^+^RORγt^+^ (**E**), IL-17^+^IFNγ^+^ (**F**) and IFN-γ^+^Tbet^+^ (**G**) expression in CD3^+^CD4^+^CD44^+^ T cells at day 6 after restimulation with indicated concentration of MOG_35–55._ Results shown are from mice pooled from two independent experiments (mean ± SD, *n* = 8 mice). Data are representative of three experiments.∗∗∗*p* < 0.001; *p* values were determined by two-way ANOVA with Sidak’s post hoc test (**A**–**G**)

To further evaluate the role of lowering cholesterol levels in CNS autoimmunity, we asked whether anti-PCSK9 could alter immune cell activation in the periphery despite the absence of differences in the clinical scores. MOG_35–55_ antigen-specific responses were compared between anti-PCSK9-treated mice and controls. Eight days after EAE immunization, splenocytes were harvested and culture with MOG_35–55_ peptide in vitro. We assessed proliferation and activation in response to MOG_35–55_ by thymidine incorporation and IL-2 secretion in the culture supernatants. In vivo treatment with anti-PCSK9 during EAE did not alter the activation and the proliferation of peripheral MOG_35–55_ specific T cells (Fig. 4A), nor IL-2 secretion (Fig. 4B). Moreover, the antigen-specific production of IL-17 (Fig. 4C) and IFN-γ (Fig. 4D) in culture supernatants was not different in anti-PCSK9 treated versus control mice. Finally, the frequency of IL-17A^+^RORγt^+^ (Fig. 4E), IL-17A^+^ IFNγ^+ ^(Fig. 4F) and Tbet^+^IFN-γ^+^ CD4^+^T cells (Fig. 4G) in activated CD4 T lymphocytes (CD3^+^CD4^+^CD44^+^) after 6 days of culture with MOG_35–55_ assessed by flow cytometry was similar in anti-PCSK9-treated mice or control mice. These results indicate that anti-PCSK9 treatment does not affect the proliferation, nor polarization of autoreactive T cells during EAE.

## Discussion

The relationship between MS pathogenesis and cholesterol homeostasis is largely debated. Previous studies reported an association between elevated levels of total circulating cholesterol and their carrier lipoproteins with a worse outcome of the disease [36–39]. However, the role of lowering cholesterol levels during MS is controversial: while statin treatments were initially proposed to be beneficial for EAE and MS, it has led to several disputed clinical studies [40]. Here we showed that the sole modulation of circulating cholesterol levels is not sufficient to impact EAE using two strategies targeting circulating cholesterol: the use of LDLr^−/−^ mice which cause a significant increase in blood cholesterol and of PCSK9 inhibitors which specifically reduces LDLr degradation and consequently lowers blood concentrations of cholesterol.

We report that elevated circulating cholesterol levels induced by LDLr deficiency do not affect the development of EAE disease and that those results are independent of the sex of the mice. Moreover, our results demonstrate that the genetic deletion of LDLr has no impact on ex vivo T cell activation, proliferation and differentiation in response to MOG_35-55_. These data indicate that the reduced clearance of lipids from the circulation in LDLr^−/−^ mice is not sufficient to substantially alter peripheral myelin-specific immune responses nor to influence EAE disease development. We propose that our observations are related to the circulating cholesterol and that they are independent of CNS cholesterol homeostasis as LDLr plays a critical role in the regulation of cholesterol metabolism outside the CNS [41]. Plasma cholesterol concentration is profoundly altered in LDLr^−/−^ mice, but does not differ significantly in any extrahepatic organ, including the brain [42–44]. Additionally, LDL uptake from the BBB does not regulate CNS cholesterol [45] and furthermore cholesterol levels in the CNS rely exclusively on local de novo cholesterol synthesis [16]. On the contrary, ApoE regulates cholesterol homeostasis within the CNS [16]. Even though LDLr deficiency has been proposed to increase murine brain ApoE levels, it did not alter brain cholesterol levels [43, 44, 46–48]. Previous studies on EAE using ApoE and LDLr-deficient mice are discordant [26–31, 49]. While both a milder and aggravated EAE has been described in ApoE-deficient mice, a protective role of LDLr deficiency has been described but only in female mice and the differences observed were subtle [27]. We do not confirm the results of this study as we show no impact of LDLr deficiency during EAE neither in female nor in male mice. We however cannot exclude that other environmental factors linked to each animal facility might explain those differences.

We then evaluated if reduction of circulating cholesterol could impact CNS autoimmunity using anti-PCSK9 antibodies, a new generation of lowering-cholesterol drug. While statins have an enzymatic inhibitory effect on cholesterol production, anti-PCSK9 monoclonal antibodies specifically reduce LDLr elimination. While we observed that anti-PCSK9 monoclonal antibodies significantly decreased the circulating cholesterol level in WT mice, the reduction of circulating cholesterol did not change the EAE clinical course nor did it have an impact on ex vivo T cell activation, proliferation and differentiation in response to MOG_35–55._ We thus demonstrate here for the first time, that an isolated decrease of total blood cholesterol levels using a monoclonal anti-PCSK9 neutralizing antibodies does not alter the adaptive immune responses during the development of CNS autoimmunity.

Those results suggest that the effects of statins on EAE are independent of their impact on circulating cholesterol. Indeed, a study applying structural equation models proposed that the benefits of simvastatin in secondary progressive MS were probably independent of circulating cholesterol [50]. Furthermore, statins could have a direct biological effect in the CNS. Interestingly, simvastatin, which is a small lipophilic molecule that can easily cross the BBB, has been proposed to inhibit CNS remyelination [25]. This would be in line with the observation that exogenous cholesterol can enter the CNS through an impaired BBB, resulting in enhanced repair and an amelioration of the neurological phenotype in two distinct models of remyelination [23]. We thus cannot exclude that lipophilic statins affect cholesterol homeostasis directly in the CNS. On the contrary, monoclonal antibodies like the anti-PCSK9 antibodies, are large hydrophilic molecules that do not have the capacity to cross the BBB, especially under conditions where BBB integrity is intact [51]. In some pathological conditions, such as diabetes, the BBB might be compromised [52]. However, even in those conditions, it is less likely that the antibodies cross the BBB [53]. Even in the unlikely presence of the anti-PCSK9 monoclonal antibodies in the CNS, it has been proposed that they do not affect brain PCSK9 levels [34]. These results suggest that the protective outcome of statins in EAE and possibly in MS are independent of their effect on lowering peripheral cholesterol. However, in contrast to statins whose pleiotropic effects have been reported including anti-inflammatory effects and immunomodulation which are beyond the decreased circulating cholesterol, relatively little is yet known about other systemic effects of anti-PCSK9 monoclonal antibodies. Furthermore, hypercholesterolemia could be beneficial for remyelination and thus anti-PSCK9 treatment could even be deleterious in EAE. We did not observe an exacerbation of EAE nor significant changes on the area of demyelination assessed by LFB/PAS staining under anti-PCSK9 treatment. However, we noted a trend towards a higher percentage of demyelination under anti-PCSK9 treatment that could even favor a deleterious role for anti-PCSK9 treatment in EAE. This remains to be investigated in the future, for example by using more specific mouse models for de- and remyelination such as the cuprizone model.

In conclusion, we demonstrate that enhancing or decreasing circulating cholesterol levels does not have an impact on EAE disease. Interestingly, a high-fat diet exacerbate EAE disease course [54, 55] but a sole high-cholesterol does not. On the contrary, a high-cholesterol diet could even dampen inflammation in EAE [23]. We thus hypothesize that MetS and not solely hypercholesterolemia impacts neuroinflammation. In a large cohort of MS patients, MetS which comprises not only dyslipidemia, but also elevated blood pressure and type 2 diabetes was positively correlated with the severity and worse outcomes in MS [56]. The control of the different components of MetS and not solely the use of lipid-lowering drugs that mainly target circulating cholesterol should be evaluated in MS. Our understanding of dyslipidemia in autoimmune disease remains incomplete. Plasma cholesterol levels currently used as traditional biomarkers for cardiovascular health have also been associated with the development of chronic diseases including autoimmune diseases but their causal contribution to disease remains unknown. Dyslipidemia and inflammation are closely interrelated and recent published work support the view that inflammation rather than blood cholesterol leads to the onset of cardiovascular diseases [57]. Thus, large-scale studies are required to investigate the relative contribution of dyslipidemia versus the other components of MetS during neuroinflammation.

## Conclusion

Our study demonstrates that circulating cholesterol does not affect the development of EAE disease. It further supports the hypothesis that statin’s beneficial effects cannot be attributed to the sole lowering serum cholesterol levels and its consequent improved hyperlipidemia, which is known to be a comorbidity in MS [58]. Nevertheless, this does not rule out that cholesterol is still an interesting target and should be further investigated especially during the recovery phase of CNS autoimmunity.

## Supplementary Information

Below is the link to the electronic supplementary material.

**Additional file 1. Figure S1.** Anti-PCSK9 treatment and LDLr^−/−^ deficiency do not significantly affect percentage of demyelination in the spinal cord of EAE mice. Histopathological staining (**A**) and quantifications of spinal cord sections (**B**) of non-immunized WT mice (NI), EAE WT mice treated with PBS or anti-PCSK9 and EAE LDLr^−/−^ mice treated with PBS. LFB/PAS staining was performed at day 16 after immunization. Five sections par mouse were quantified (n=3). Scale bars 500μm (top panels), 100μm (bottom panels). NS, not significant; *p* values were determined by unpaired Student’s *t* test.

## Data Availability

Further information and requests for resources and reagents should be directed to and will be fulfilled by the Lead Contact, Caroline Pot (Caroline.Pot-Kreis@chuv.ch). This study did not generate new unique reagents.

## Abbreviations

MS: Multiple sclerosis
CNS: Central nervous system
TG: Triglycerides
tChol: Total cholesterol
HDLc: High-density lipoprotein cholesterol
LDLc: Low-density lipoprotein cholesterol
EAE: Experimental autoimmune encephalomyelitis
RRMS: Relapsing remitting MS
PMS: Secondary progressive MS
ApoE: Apolipoprotein E
PCSK9: Proprotein convertase subtilisin/kexin type 9
BBB: The blood–brain barrier
MOG: Myelin oligodendrocyte glycoprotein
