# Supplementary material for: Lowering blood cholesterol does not affect neuroinflammation in experimental autoimmune encephalomyelitis
Source: J Neuroinflammation. 2022 Feb 7;19:42. doi: 10.1186/s12974-022-02409-x (PMC8822860; doi:10.1186/s12974-022-02409-x)
Supplement: Supplementary file 1 — Additional file 1. Figure S1. Anti-PCSK9 treatment and LDLr−/− deficiency do not significantly affect percentage of demyelination in the spinal cord of EAE mice. Histopathological staining (A) and quantifications of spinal cord sections (B) of non-immunized WT mice (NI), EAE WT mice treated with PBS or anti-PCSK9 and EAE LDLr−/− mice treated with PBS. LFB/PAS staining was performed at day 16 after immunization. Five sections par mouse were quantified (n=3). Scale bars 500μm (top panels), 100μm (bottom panels). NS, not significant; p values were determined by unpaired Student’s t test. [file 12974_2022_2409_MOESM1_ESM.pdf]

Supplementary Figure 1

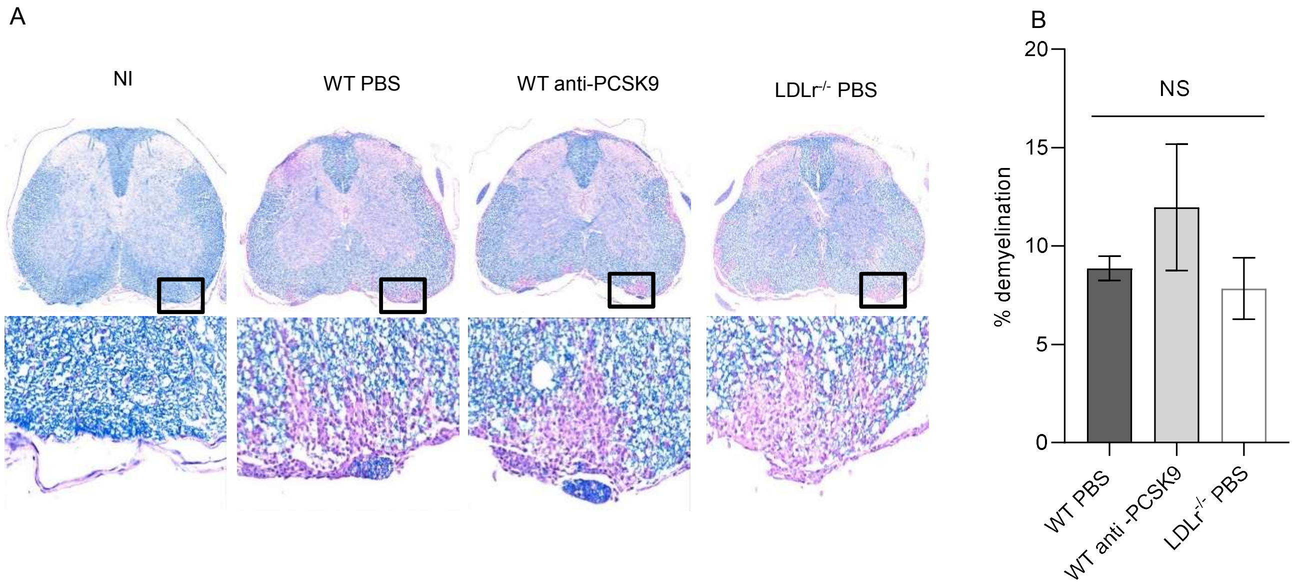

Figure S1 **Anti-PCSK9 treatment and LDLr$^{-/-}$ deficiency do not significantly affect percentage of demyelination in the spinal cord of EAE mice.** Histopathological staining (A) and quantifications of spinal cord sections (B) of non-immunized WT mice (NI), EAE WT mice treated with PBS or anti-PCSK9 and EAE LDLr$^{-/-}$ mice treated with PBS. LFB/PAS staining was performed at day 16 after immunization. Five sections par mouse were quantified (n=3). Scale bars 500µm (top panels), 100µm (bottom panels). NS, not significant; p values were determined by unpaired Student’s t test.
